# Evaluation of Antiulcer and Cytotoxic Potential of the Leaf, Flower, and Fruit Extracts of* Calotropis procera* and Isolation of a New Lignan Glycoside

**DOI:** 10.1155/2017/8086791

**Published:** 2017-08-30

**Authors:** Areej Mohammad Al-Taweel, Shagufta Perveen, Ghada Ahmed Fawzy, Attiq Ur Rehman, Afsar Khan, Rashad Mehmood, Laila Mohamed Fadda

**Affiliations:** ^1^Department of Pharmacognosy, College of Pharmacy, King Saud University, P.O. Box 2457, Riyadh 11451, Saudi Arabia; ^2^Department of Pharmacognosy, Faculty of Pharmacy, Cairo University, Cairo 11562, Egypt; ^3^Department of Chemistry, COMSATS Institute of Information Technology, Abbottabad 22060, Pakistan; ^4^Department of Chemistry, University of Education, Vehari Campus, Vehari 61100, Pakistan; ^5^Department of Pharmacology, College of Pharmacy, King Saud University, P.O. Box 2457, Riyadh 11451, Saudi Arabia

## Abstract

*Calotropis procera* is traditionally used for treating many diseases including ulcers and tumors. It was thus deemed of interest to investigate and compare the antiulcer and cytotoxic activities of* C. procera *leaf, flower, and fruit extracts in an attempt to verify its traditional uses. Phytochemical studies on the fruits, flowers, and leaves of* C. procera,* collected from the desert of Saudi Arabia, led to the isolation of one new lignan 7′-methoxy-3′-*O*-demethyl-tanegool-9-*O*-*β*-d-glucopyranoside and five known compounds from the flowers, four compounds from leaves, and a flavonoid glycoside and a lignan glycoside from the fruits. The structures of compounds were determined by spectroscopic techniques. Ethanol extracts of the three parts of* C. procera *were evaluated for their antiulcer activity and we found that the leaf extract possessed a powerful antiulcer activity which could be considered as a promising drug candidate. All the extracts and the isolated compounds were evaluated for their cytotoxic activity against MCF-7, HCT-116, HepG-2, and A-549 human cancer cell lines. Compound** 2** was highly active on all the cell lines, whereas compounds** 5** and** 11** were more selective on colon and liver cell lines. Compound** 10** demonstrated a significant activity on liver and lung cancer cell lines.

## 1. Introduction

*Calotropis procera* (Cp) is a xerophytic perennial shrub native to tropical and subtropical Africa and Asia and common in the Middle East [1]. It is known in Arabic as Ushar, found in northern and southern Hijaz, in the central region, and in southern and northeastern regions of Saudi Arabia. Different parts of Cp had been widely used in traditional medicine due to its pharmacologically active compounds found in all the plant's parts, flowers, roots, and leaves, and its milky latex which is an exudate from damaged and broken leaves and stems. The decoction of the aerial parts of Cp is commonly used in Saudi Arabian traditional medicine for the treatment of a variety of diseases including fever, tumors, joint pain, ulcer, muscular spasm, and constipation [1–3]. Different extracts of the plant showed significant antipyretic, analgesic, anticancer, antibacterial, nematocidal [4], larvicidal [5], and neuromuscular blocking activities [2]. The whole parts of Cp have been investigated previously for its phytoconstituents, which revealed the presence of cardenolides, anthocyanins, and triterpenoids [6].

Gastric ulcer is a widespread gastrointestinal disorder and a multietiological illness that influences an extensive population worldwide. Hence, there is a strong need to develop new natural drugs from herbs and plants for the treatment of this disease. Previous antiulcer studies on different parts of Cp included work on the chloroform fraction of Cp root extract, which showed significant antiulcer effect in guinea pigs [7]; latex extracts of Cp gave a protective effect on experimentally induced gastric ulcers in rats [8] while the stem bark of the plant demonstrated a strong antiulcer activity in two acute models: aspirin and ethanol in albino rats [9]. Additionally, a recent US patent proved that the extract of aerial parts of Cp possessed potent antiulcerative colitis activity [10].

In the course of our ongoing research towards the isolation of biologically active constituents from Saudi medicinal plants, either cultivated or wild, having chemical constituents with cytotoxicity, we had the opportunity to work on the fruits, flowers, and leaves of Cp growing in Saudi Arabia to investigate its secondary metabolites and their biological activities. Our aim in this study was to find out the extract which has strong antiulcer activity among flowers, fruits, and leaves. We also focused to get the most promising cytotoxic extract and compounds from this plant. As a result of our phytochemical studies on fruits, flowers, and leaves of Cp, we successfully isolated one new lignan 7′-methoxy-3′-*O*-demethyl-tanegool-9-O-*β*-d-glucopyranoside (**1**) and five known compounds, methyl ferulate (**2**), rosmarinic acid (**3**), methyl rosmarinate (**4**), methyl 4-*O*-*β*-d-glucopyranosyl ferulate (**5**), and pinoresinol 4-*O*-glucoside (**6**), for the first time from the flowers; four known compounds, *β*-sitosterol (**7**), lupeol (**8**), lupeol-3-*O*-acetate (**9**), and calotropagenin (**10**), from the leaves; and the flavonoid glycoside kaempferol 3-*O*-*α*-l-rhamnopyranosyl-(1→6)-*β*-d-glucopyranoside (**11**) and syringaresinol 4-*O*-glucoside (**12**) for the first time from the fruits. Furthermore, we studied the antiulcer effect of Cp (leaves, flowers, and fruits) and we found that the leaves and flowers extracts exhibited significant antiulcerogenic effect on male albino rats. All the extracts and the isolated compounds were also evaluated for their cytotoxic activity against MCF-7, HCT-116, HepG-2, and A-549 human cancer cell lines and we found that compound** 2 **showed significant activity on all the tested cancer cell lines, while compounds** 5 **and** 11** exhibited strong activity against colon and liver cancer cell lines. Compound** 10** exhibited significant activity on liver and lung cancer cell lines. This is our first report to test the cytotoxicity of these compounds on different human cancer cell lines.

## 2. Materials and Methods

### 2.1. Plant Material

The leaves, flowers, and fruits of Cp were collected individually in early March 2015 from the desert area of Riyadh city, Saudi Arabia, and identified by the taxonomist Dr. M. Atiqur Rahman, College of Pharmacy, Medicinal, Aromatic and Poisonous Plants Research Center, King Saud University. A voucher specimen (12491) has been kept in the herbarium of the College of Pharmacy, King Saud University.

### 2.2. Extraction and Isolation

The shade-dried leaves, flowers, and fruits (0.5 kg each) were extracted with ethanol (5 × 3 L) at room temperature. The ethanolic extracts of each part were evaporated under reduced pressure to obtain a thick gummy material, 50 g, 65 g, and 75 g from leaf, flower, and fruit, respectively.

Flower extract was dissolved in water and concentrated to 60 mL and chromatographed on Sephadex LH-20 column (4 × 70 cm, flow rate: 50 mL/h) using H_2_O and mixtures of H_2_O/MeOH (9 : 1–1 : 1) to obtain six fractions and then these were further combined into three major subfractions FL1–FL3 due to their similarity on TLC. The fraction FL1 was rechromatographed on Sephadex LH-20 column (2 × 40 cm, flow rate: 20 mL/h) using 1 : 1 H_2_O/MeOH to give compounds** 5** (20 mg) and** 6** (3 mg). Fraction FL2 was subjected to Sephadex LH-20 column (2 × 40 cm, flow rate: 20 mL/h) using 6 : 4 H_2_O/MeOH and yielded compound** 1** (15 mg). Fraction FL3 was subjected to silica gel column (2 × 40, flow rate: 60 mL/h) using 8.5 : 1.5 CHCl_3_/MeOH and yielded compounds** 2** (20 mg),** 3** (4 mg), and** 4** (5 mg). A part of EtOH soluble leaves extract was subjected to silica gel column chromatography (4 × 70 cm, flow rate: 150 mL/h) eluting with CHCl_3_ (100%) and CHCl_3_/MeOH (5%, 10%, and 15%) in an increasing order of polarity to give five fractions which were combined into three subfractions L1–L3 due to their similar spots on TLC. Fraction L1 was rechromatographed on silica gel column (2 × 40 cm, flow rate: 100 mL/h) using CHCl_3_/MeOH (9.5 : 0.5) as eluent to obtain compound** 7** (7 mg). Fraction L2 was loaded on silica gel column (2 × 40 cm, flow rate: 80 mL/h) eluting with 9.3 : 0.7 CHCl_3_/MeOH and it yielded compounds** 9** (5 mg) and** 8** (6 mg) from top and tail fractions, respectively. Fraction L3 was rechromatographed over silica gel column (2 × 40 cm, flow rate: 80 mL/h) using 9.0 : 1.0 CHCl_3_/MeOH as eluent to obtain compound** 10** (15 mg). A part of the ethanol soluble fraction of Cp fruits was chromatographed on Sephadex LH-20 column (2 × 40 cm, flow rate: 20 mL/h) using H_2_O/MeOH (1 : 1) as eluent to get compounds** 11** (20 mg) and** 12** (5 mg).

### 2.3. Phytochemical and Spectroscopic Procedures

Optical activity was measured on a JASCO P-2000 polarimeter (JASCO Corporation, 2967-5, Tokyo, Japan). The IR spectra were recorded on 460 Shimadzu spectrometer. The ^1^H, ^13^C NMR, and 2D NMR spectra were recorded on a Bruker AMX-500 spectrometer and tetramethylsilane (TMS) was used as an internal standard. Chemical shifts are given in ppm (*δ*) relative to tetramethylsilane as an internal standard and scalar coupling constants (*J*) are reported in Hertz. ESI-MS analyses were measured on an Agilent Triple Quadrupole 6410 QQQ LC/MS mass spectrometer with ESI ion source (gas temperature 350°C, nebulizer pressure 60 psi, and gas flow rate 10 L/min) operating in the negative and positive scan ionization modes through direct infusion method using H_2_O/MeOH (1 : 1 v/v) with flow rate of 0.3 mL/min. Column chromatography was carried out on Sephadex LH-20 (Merck, Darmstadt, Germany). Thin layer chromatography was performed on precoated TLC plates (RP-18 F_254_, Merck, Germany); the detection was done at 254 and 354 nm and by spraying with ceric sulphate and sulphuric acid reagents.

#### 2.3.1. Chemicals

Alcian Blue 8GX (chloromethylated copper phthalocyanine-thiourea reaction products), Crystal Violet (hexamethyl-*p*-rosaniline chloride), Ellman's reagent (5,5′-dithiobis(2-nitrobenzoic acid)), magnesium chloride, sodium chloride, and Sephadex LH-20 were purchased from Sigma-Aldrich (St. Louis, MO, USA). Ceric sulphate, ethanol, methanol,* n*-butanol, chloroform, ethyl acetate, acetic acid, hydrochloric acid, trichloroacetic acid, sodium hydroxide, and silica gel were purchased from Merck (Darmstadt, Germany).

### 2.4. Compound** 1**

Yellow amorphous powder (15.0 mg); [*α*]^25^_D_ 15.5 (*c* 0.1, MeOH); UV: *λ*_max_ (MeOH): 220 (4.33), 264 (4.18), 290 (3.65) nm; IR (KBr) *ν*_max_ 3397, 1708, 1510, 1455 cm^−1^; ESI-MS (+)* m/z*: 538. ^1^H and ^13^C (500, 125 MHz, DMSO-*d6*) NMR data (see Table 1), for COSY and HMBC (see Figure 2).

### 2.5. Acid Hydrolysis of Compound** 1**

A methanolic solution (6 mL) of compound** 1** (3 mg) was mixed with 1 N HCl (4 mL) and refluxed for 2 h. Then, the solution was concentrated under reduced pressure, diluted with H_2_O (8 mL), and extracted with EtOAc to obtain lignan. The sugar was obtained from the aqueous part and identified as d-glucose by TLC system using BuOH : AcOH : H_2_O (4 : 1 : 5) as a mobile phase and ceric sulphate as a spraying agent with heating at 130°C on a hot plate.

### 2.6. Antiulcer Activity

#### 2.6.1. Animals and Dosing

Male albino rats (120–150 g) of Sprague-Dawley strain were obtained from the Experimental Animal Center, Faculty of Pharmacy, King Saud University, Saudi Arabia. The animal experimental protocol was approved by the Animal Care and Ethical Committee of the Faculty of Pharmacy, King Saud University. Animals were housed in clean acrylic cages and maintained under standard conditions (12-hour light/12-hour dark cycle) at a controlled temperature at 20–22°C and 60% humidity. Rats were fed a standard rat pellet diet with free access to tap water ad libitum for 1 week for acclimatization. After 1 week of acclimation, the rats were made to fast overnight before treatment and were divided randomly into eight groups, each of six rats. Group I was the control group. Group II was given ranitidine at a dose of 50 mg/kg p.o. Groups III–VIII were treated with two doses, 200 and 400 mg/kg, of each of the total ethanol extracts of Cp leaves, flowers, and fruits, orally in the antiulcer study and i.p. for gastric secretion evaluation.

#### 2.6.2. Pylorus-Ligated Rats

Before pylorus ligation, rats were fasted for 36 h under ether anesthesia. Cp extracts were administered i.p. immediately after pylorus ligation (Shay). The rats were sacrificed 6 h after pylorus ligation. The stomachs were taken, the contents were collected, and volumes were measured, centrifuged, and evaluated for titratable acidity against 0.01 mol/L NaOH at pH 7 [11].

#### 2.6.3. Gastric Lesions Induced by Different Ulcerogens

Cp extracts in all doses in question were given 30 min before the administration of the ulcerogens. Each rat was given 1 mL of each ulcerogen (80% ethanol, 0.2 mol/L NaOH, or 25% NaCl). After 24 h, the rats were sacrificed and the stomachs were removed and opened along the greater curvature. After washing with saline solution, they were inspected for lesions and scoring in the stomach. The scoring of stomach lesions was as follows: patchy lesions of the stomach induced by ethanol were scored according to the following scale: 0 = normal mucosa; 1 = hyperemic mucosa or up to 3 small patches; 2 = from 4 to 10 small patches; 3 = more than 10 small or up to 3 medium-sized patches; 4 = from 4 to 6 medium-sized patches; 5 = more than 6 medium-sized or up to 3 large patches; 6 = from 4 to 6 large patches; 7 = from 7 to 10 large patches; 8 = more than 10 large patches or extensive necrotic zones. “Small” was defined as up to 2 mm across (max. diameter), “medium-sized” between 2 and 4 mm across, and “large” more than 4 mm across [12].

#### 2.6.4. Indomethacin-Induced Gastric Lesions

The ethanol extracts of Cp leaf, flower, and fruit were given to rats 30 min before indomethacin administration at two doses of 200 and 400 mg/kg. Indomethacin was orally administered to the 36 h fasted rats (30 mg/kg). The animals were sacrificed 6 h after indomethacin treatment; stomachs were removed, washed with normal saline, and then inspected for ulceration [13].

#### 2.6.5. Hypothermic Restraint Stress-Induced Ulcers

The animals were fasted (36 h) but had access to water. Thirty minutes after the oral administration of the different doses of Cp extracts, the rats were relocated to restraint cages and sited inside a ventilated refrigerator kept at 3 ± 1°C for 3 h. The animals were sacrificed and the stomachs were removed and inspected for ulceration and the severity of intraluminal bleeding according to the method described by Chiu et al. [14].

#### 2.6.6. Gastric Wall Mucus (GWM) Estimation

GWM was estimated according to the modified procedure of Corne et al. [15]. The glandular segment of the stomach was separated from the rumen, weighed, and transferred immediately to 10 mL of 0.1% w/v Alcian Blue solution. Tissue was stained for 2 h in Alcian Blue, initially after 15 min and then after 45 min. Dye complexed with the gastric wall mucus was extracted with 10 mL of 0.5 mmol/L magnesium chloride. The resulting emulsion was centrifuged at 4000 rpm for 10 min and the absorbance of the aqueous layer was recorded at 580 nm. The quantity of Alcian Blue extracted per gram of wet glandular tissue was then calculated.

#### 2.6.7. Estimation of Reduced Glutathione

The glandular part of the stomach was homogenized and centrifuged at 3000 rpm. Two milliliters of supernatant was mixed with 4 mL of 0.4 mol/L Tris buffer at pH 8.9. A 0.1 mL Ellman's reagent was added and the absorbance was measured within 5 min at 412 nm [16].

#### 2.6.8. Determination of Malondialdehyde (MDA)

1 h after ethanol administration, the animals were killed. The stomachs were removed, washed, and homogenized, and 1.25 mL of 20% trichloroacetic acid (TCA) was mixed with 250 *μ*L of stomachs homogenate. The mixture was heated for 30 min in a boiling water bath and then cooled and centrifuged for 10 minutes at 4°C. The absorbance of the developed pink-colored product was measured at 535 nm. MDA concentration was expressed as nmol/g of tissue [17].

#### 2.6.9. Histopathological Study

Small pieces of stomachs were fixed by 4% neutral buffer formalin and 0.1 M phosphate buffer (pH 7.4) and then embedded into paraffin, sectioned to 5-6 *μ*m thick pieces, and mounted on glass microscope slides using standard histopathological techniques. The sections were stained with Hematoxyline and Eosin stain and examined under a light microscope [18]. The slides were examined microscopically for pathomorphological changes such as congestion, hemorrhage, edema, and erosions using an arbitrary scale for severity assessment of these changes.

#### 2.6.10. Statistical Analysis

Values in tables and figures are given as mean ± SE. Data were analyzed using one-way analysis of variance (ANOVA) followed by Student's* t*-test.

### 2.7. Cytotoxicity Assay

#### 2.7.1. Cell Culture

Human cancer cell lines (MCF-7 cells (breast cancer cell line), HCT-116 (colon carcinoma), HepG-2 (hepatocellular carcinoma), and A-549 (human lung carcinoma)) were obtained from the Holding Company for Biological Products and Vaccines (VACSERA) Tissue Culture Unit. Cells were cultured in Dulbecco's modified Eagle's medium (DMEM) including 10% heat-inactivated fetal bovine serum, HEPES buffer, 1% L-glutamine, and 50 *μ*g/mL gentamycin. The cells were kept at 37°C under humidified conditions with 5% CO_2_ and were subcultured twice a week.

#### 2.7.2. Evaluation of Cytotoxicity Using the Viability Assay

The cells were seeded in a 96-well plate at a concentration of 1 × 10^4^ cells per well in 100 *μ*L of growth medium. Different concentrations of the test samples were added after 24 h of seeding in fresh medium. Serial twofold dilutions of the tested samples were added to confluent cell monolayers dispensed into 96-well microtiter plates (Falcon, NJ, USA). The microtiter plates were incubated at 37°C for 48 h. Three wells were used for each concentration of the test sample. Control cells were incubated without test sample and with or without DMSO. By the end of the incubation period, media were aspirated and Crystal Violet solution (1%) was added to each well for 30 min. Glacial acetic acid (30%) was then added to all wells and mixed properly. Colorimetric evaluation of fixed cells was performed by taking absorbance measurements using an automatic microplate reader (TECAN, Inc.) at 490 nm. The treated samples were compared with control cells in the absence of tested samples. The optical density was measured to determine the number of viable cells. The percentage of viability was calculated as described previously [19], while 50% inhibitory concentration (IC_50_) was determined from graphic plots of the dose response curve using GraphPad Prism software (San Diego, CA, USA). Vinblastine sulfate was used as a reference drug [20].

#### 2.7.3. Statistical Analyses

Data were expressed as means ± SD. For multivariable comparisons, one-way ANOVA was conducted, followed by Tukey-Kramer testing using the GraphPad InStat (ISI Software) computer program. Differences were considered significant at *p* < 0.05.

## 3. Results

**Figure 1:**
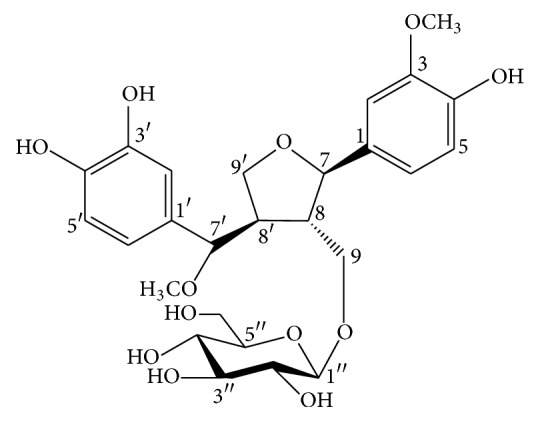
Chemical structure of compound** 1**.

Chromatographic separation of the chloroform and* n*-butanol fractions of ethanol extract of Cp on silica gel, Sephadex LH-20, and RP-18 silica yielded one new lignan 7′-methoxy-3′-*O*-demethyl-tanegool-9-*O*-*β*-d-glucopyranoside (**1**) (Figure 1) and five known compounds, methyl ferulate (**2**), rosmarinic acid (**3**), methyl rosmarinate (**4**) [21], methyl 4-*β*-d-glucopyranosyl-ferulate (**5**) [22], and pinoresinol 4-*O*-glucoside (**6**) [23] from flowers; four known compounds, *β*-sitosterol (**7**), lupeol (**8**), lupeol-3-*O*-acetate (**9**) [24], and calotropagenin (**10**) [25], from leaves; and flavonoid glycoside kaempferol 3-*O*-*α*-l-rhamnopyranosyl-(1→6)-*β*-d-glucopyranoside (**11**) and syringaresinol 4-*O*-glucoside (**12**) from the fruits. Their structures were elucidated by spectroscopic data analysis, including IR, 1D and 2D NMR, and HRESIMS.

### 3.1. Structure Elucidation of New Compound

**Figure 2:**
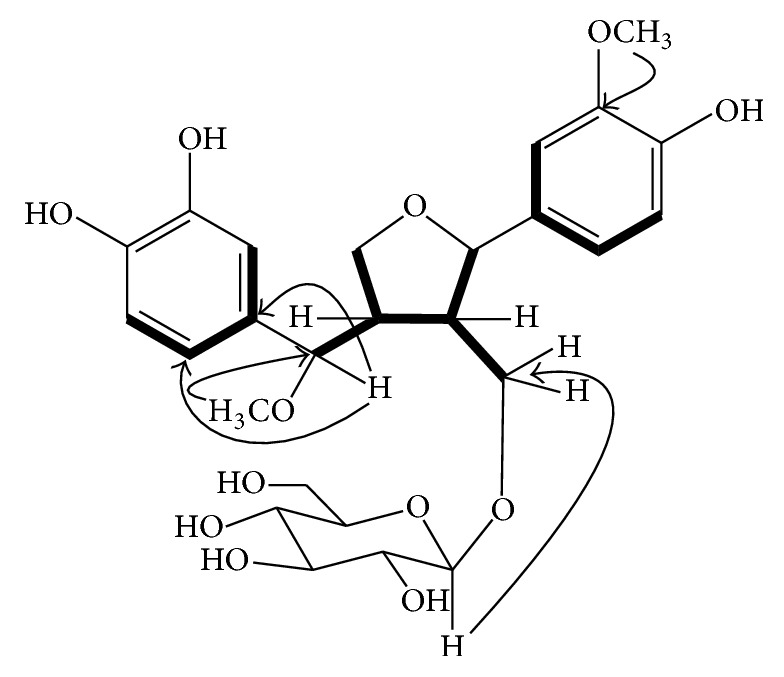
Key HMBC (arrows) and ^1^H-^1^H COSY (thick lines) correlations of compound** 1**.

Compound** 1** was obtained as a yellow gummy material. The molecular formula C_26_H_34_O_12_ was established by the observation of molecular ion peak at* m/z* 538 in positive ESI-MS. The IR spectrum indicated the absorption bands for hydroxyl groups at 3397, carbonyl group at 1708, and aromatic rings at 1455 and 1510 cm^−1^. The ^1^H NMR spectrum of** 1** showed signals of two ABX ring systems at *δ*_*H*_ 6.76, 6.88, 6.93, 7.00, 7.15, and 8.16, an anomeric proton at *δ*_*H*_ 4.94, two methoxy groups at *δ*_*H*_ 3.83 and 3.84, protons signals at *δ*_*H*_ 3.75, 3.89, and 4.18 for three oxygenated methylenes, two oxygenated methine protons signals at *δ*_*H*_ 4.67 and 4.71, and two nonoxygenated methine protons signals at *δ*_*H*_ 3.05, indicating the presence of lignan skeleton attached with a sugar moiety. The ^13^C NMR spectrum showed signals of two aromatic rings, a *β*-glucopyranosyl sugar moiety, two methoxy groups along with two methylene carbons at *δ*_*C*_ 71.32 and 71.37, two oxygen bearing methine carbon signals at *δ*_*C*_ 85.6 and 85.9, and one upfield methine carbon at *δ*_*C*_ 53.8 (Table 1). These spectroscopic data were closely similar to the previously reported compound 3′-*O*-demethyl-tanegool isolated from* Taxus mairei* [23] except for the presence of methoxy group at C-7′ instead of a hydroxyl group, the absence of a methoxy group at C-3′ (but the presence of a hydroxy group), and the presence of a sugar moiety at C-9 hydroxymethylene carbon. The point of attachment of sugar moiety with the lignan unit was confirmed by the HMBC correlations of *δ*_*H*_ 4.94 (anomeric, H-1′′) to *δ*_*C*_ 71.37 (lignan C-9) (Figure 2). The oxygenated quaternary carbons of the aromatic rings appeared at *δ*_*C*_ 149.3, 147.7, 145.9, and 145.7 indicating the presence of one methoxy and three hydroxy groups at the rings. One of the methoxy groups was attached to the methine carbon of the lignan skeleton [26]. This was confirmed through the HMBC correlations of *δ*_*H*_ 3.83 (7′-OCH_3_) to *δ*_*C*_ 85.9 (lignan C-7′), while the signal of methoxy group at *δ*_*H*_ 3.84 (3-OCH_3_) showed correlations with *δ*_*C*_ 149.3 (C-3). Acid hydrolysis of compound** 1** yielded d-glucose, identified by the sign of optical rotation and by co-TLC with authentic standard sample. All the above evidence suggested the existence of 7′-methoxy-3′-*O*-demethyl-tanegool-9-O-*β*-d-glucopyranoside identified as** 1**.

### 3.2. Antiulcer Activity

Concerning gastric secretion, titratable acidity, and gastric lesion index in pylorus Shay rats, all the Cp extracts exhibited a significant decrease in the aforementioned parameters except the fruit extracts at the two doses with respect to gastric secretion and the flowers extract at 200 mg/kg toward gastric lesion index (Table 2). Cp ethanol leaf, flower, and fruit extracts significantly lessened the ulcer index induced by ethanol. On the other hand, only the leaf and flower extracts reduced ulcer index induced by either sodium hydroxide or sodium chloride. Cp leaf extract (400 mg/kg) showed the most pronounced antiulcer effect (Table 3). Data in Table 4 confirmed that only Cp leaf extract, at a dose of 400 mg/kg, exhibited a significant decline in ulcer index induced by indomethacin while the fruits and flower extracts were ineffective towards reducing ulcer index in this concern.

**Figure 3:**
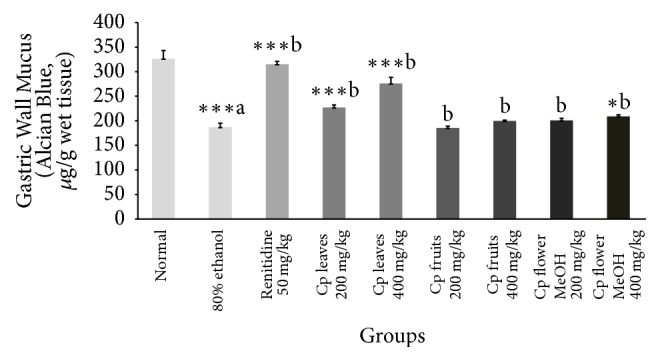
Effect of Cp extracts on the changes in gastric wall mucus induced by 80% ethanol. Six rats were used in each group. ^*∗*^*p* < 0.05, ^*∗∗∗*^*p* < 0.001 versus control (80% ethanol only) group, Student's* t*-test, (a) as compared to the control group and (b) as compared to the 80% ethanol only group.

**Figure 4:**
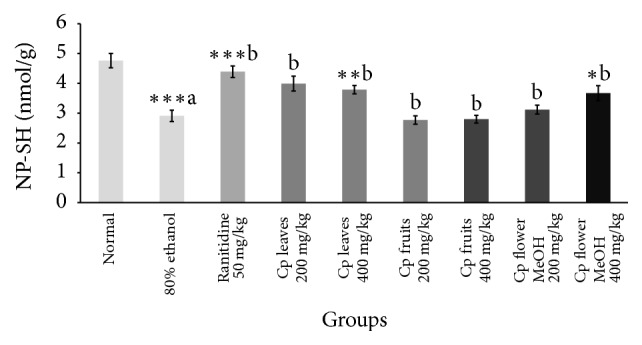
Effect of Cp extracts on GSH concentration in gastric ulcer induced by 80% ethanol. Six rats were used in each group. ^*∗*^*p* < 0.05, ^*∗∗*^*p* < 0.01, and ^*∗∗∗*^*p* < 0.001 versus control (80% ethanol only) group, Student's* t*-test, (a) as compared to the control group and (b) as compared to the 80% ethanol only group.

**Figure 5:**
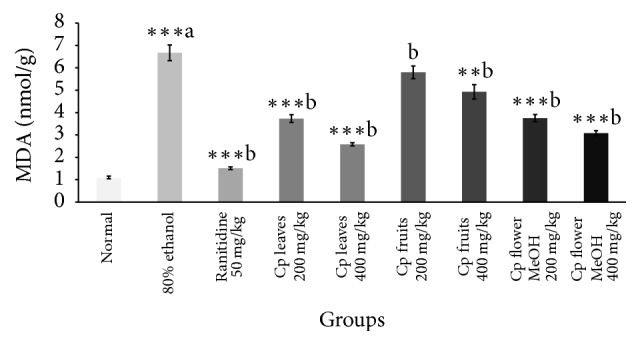
Effect of Cp extracts on MDA concentration in gastric ulcer induced by 80% ethanol. Six rats were used in each group. ^*∗∗*^*p* < 0.01, ^*∗∗∗*^*p* < 0.001 versus control (80% ethanol only) group, Student's* t*-test, (a) as compared to the control group and (b) as compared to the 80% ethanol only group.

Data represented in Table 5 confirmed that both intraluminal bleeding score and gastric lesion ulcer index were significantly reduced by the administration of only leaf and flower extracts. Ethanol decreased the gastric wall mucus in the stomach tissue compared to normal control (Table 5). Treatment with Cp leaf extract, at the two doses, exhibited the most significant progress in gastric wall mucus (Figure 3). Moreover, 80% ethanol induced a significant increase in MDA (Figure 5) level while glutathione and protein levels were decreased. The Cp leaf and flower extracts exhibited a highly significant decline in MDA and an increase in total protein levels, while only leaf and flower extracts at 400 mg/kg upregulated glutathione level (Figures 4 and 5).

**Figure 6:**
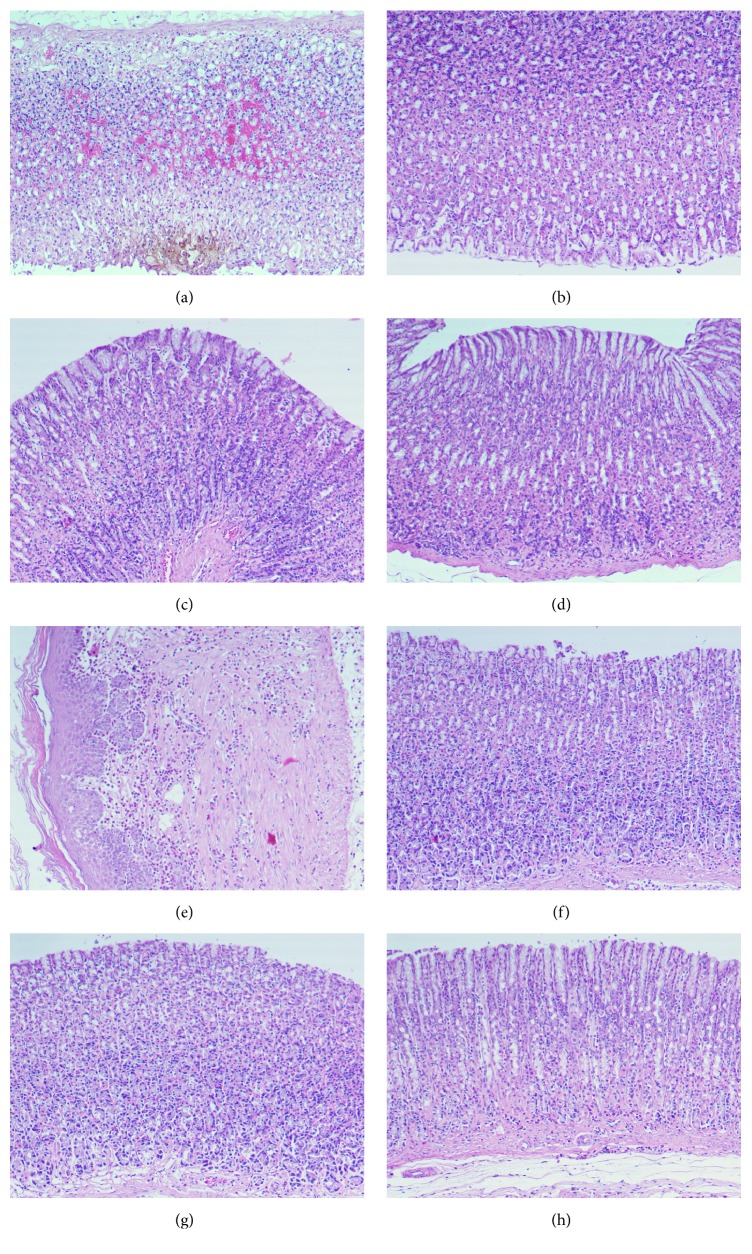
Light micrographs illustrating the effect of Cp extracts on ethanol-induced gastric lesions in rats. (a) Ethanol-induced gastric mucosal necrosis and congestion. (b) Pretreatment of rats with ranitidine, 50 mg/kg. (c) Pretreatment of rats with Cp leaves, 200 mg/kg. (d) Pretreatment with Cp leaves, 400 mg/kg. (e) Pretreatment with Cp fruits, 200 mg/kg. (f) Pretreatment with Cp fruits, 400 mg/kg. (g) Pretreatment with Cp flowers, 200 mg/kg. (h) Pretreatment with Cp flowers, 400 mg/kg.

Histopathological examination using H&E demonstrated ethanol-induced gastric mucosal congestion and necrosis. Pretreatment of rats with Cp leaf extract, 200 mg/kg and 400 mg/kg, ameliorated the congestion and necrosis induced by ethanol (Figure 6).

### 3.3. Cytotoxicity

The cytotoxic activity of total ethanol extract of Cp leaf, flower, and fruit as well as compounds** 1**,** 2**,** 5**,** 10**, and** 11** was studied using CVS method and vinblastine sulphate was used as a reference drug. Four human cancer cell lines were employed in this study, and IC_50_ was calculated for each cell line. From the cytotoxicity results the three ethanol extracts of Cp possessed moderate cytotoxic activities, being more selective on colon and liver carcinomas (Table 6). The isolated compounds demonstrated more promising cytotoxic activity. Compound** 2** was highly active on all the cell lines (IC_50_ ranging from 4.99 to 12.60 *μ*g/mL), whereas compounds** 11** and** 5** were more selective on colon and liver cell lines (IC_50_: 6.16, 11.6, 3.49, and 7.19 *μ*g/mL, resp.). Compound** 10** showed significant activity on liver and lung cancer cell lines (IC_50_: 10.40 and 6.50 *μ*g/mL, resp.), while the new lignan** 1** was not active on any cancer cell line.

## 4. Discussion

Based on our study, Cp extracts, especially the leaf extract, possessed a powerful cytoprotective effect as well as significant antisecretory and antiulcer activities. This is in addition to their ability to cause a decline in the titratable acidity and lesions in pylorus-ligated Shay rats as well as reduction in the basal gastric secretion volume. Herein, Cp leaf extract produced significant dose-dependent gastric mucosal protection in ulcers induced by different ulcerating agents including ethanol and sodium hydroxide. Gastric ulcers induced by ethanol have been used as a model for the assessment of gastroprotecting power [27]. Ethanol-induced gastric lesions are associated with a decrease in the gastric mucus, accompanied with excessive creation of superoxide anions and hydroperoxy free radicals, hence increasing lipid peroxide production which sequentially harms cells and injures cell membranes [28]. The cytoprotective effect of Cp leaf extract may be due to its ability to reduce gastric acid secretion and/or augment the mucosal defensive factors as well as inhibit lipid peroxide liberation [29]. Pretreatment with Cp leaf extract exhibited marked protection against indomethacin-induced stomach injury. The level of gastric mucosal lipid peroxidation products is directly proportional to stomach injury in cold restraint-stressed rats [30]. Cp leaf extract demonstrated a protecting effect on cold restraint stress possibly by its antioxidant activity. Moreover, Cp leaf extract markedly preserved gastric mucosa and the basic epithelium against harmful effects of ulcerating agents such as alkalis and indomethacin.

A marked depletion in gastric glutathione after ethanol administration is attributed to excessive liberation of oxygen derived free radicals that disturb gastric integrity [31–33]. Herein, Cp leaf extract produced a dose-dependent increase in GSH (Figure 4) and a marked decline in lipid peroxide level and effected mucosal protection. The antiulcer activity of Cp is possibly due to its antioxidant power and by fortifying the mucosal barrier, which is the initial line that guards against internal and external ulcerogenic agents. This promising effect may be attributed to the phytoconstituents of each extract.

Concerning the cytotoxic activity, compound** 1** did not show activity on the tested cell lines, while methyl ferulate (**2**) was highly active on all four cancer cell lines. This is in agreement with the literature [34]. According to previous studies, methyl ferulate demonstrated potential anti-inflammatory activity in lipopolysaccharide activated macrophage cells and was also proven to be equally active to ferulic acid with respect to antioxidant activity [35, 36]. Compounds** 5** and** 11** demonstrated selective cytotoxic activity on colon and liver cell lines in our study. This could be considered as the first report on cytotoxic activity of compound** 5**, yet, in another investigation, compound** 11** did not demonstrate cytotoxic effect on SK-MEL, KB, BT-549, and SK-OV-3 human cancer cell lines [37]; hence, we can suggest that colon and liver cell lines are sensitive to this compound. Compound** 10** showed significant activity on liver and lung cancer cell lines, which is in agreement with previous investigations [38].

In conclusion, the obtained data established that Cp leaf extract has a powerful antiulcer activity and may be considered as a promising drug candidate or could be supplied with other antiulcer drugs to decrease their side effects and increase their bioavailability. Moreover, cytotoxicity results augment the biological significance of the isolated phytoconstituents and provide the basis for more detailed pharmacological investigations.

## Supplementary Material

Fig. 1: ^13^C NMR spectrum of compound **1**.
Fig. 2: Expanded ^13^C NMR spectrum of compound **1**.
Fig. 3: DEPT 135 NMR spectrum of compound **1**.
Fig. 4: Expanded DEPT 135 NMR spectrum of compound **1**.
Fig. 5: DEPT 90 NMR spectrum of compound **1**.
Fig. 6: ^1^H NMR spectrum of compound **1**.
Fig. 7: Expanded ^1^H NMR spectrum of compound **1**.
Fig. 8: HMBC spectrum of compound **1**.
Fig. 9: HSQC spectrum of compound **1**.
Fig. 10: ESI-MS (+ve) spectrum of compound **1**.

## Figures and Tables

**Table 1:** ^1^H (500 MHz) and ^13^C (125 MHz) NMR spectroscopic data of compound **1** (DMSO-*d6*).

Position	*δ* (H)	*δ* (C)
1	—	136.0
2	8.16 (m)	118.5
3	—	149.3
4	—	147.7
5	7.15 (d, 5.0)	116.4
6	6.93 (s)	109.7
7	4.71 (br s)	85.6
8	3.05 (br s)	54.0
9	4.18 (br d)	71.37
1′	—	132.4
2′	7.00 (s)	110.2
3′	—	145.7
4′	—	145.9
5′	6.88 (d, 5.0)	114.9
6′	6.76 (m)	118.7
7′	4.67 (br s)	85.9
8′	3.05 (br s)	53.8
9′	3.75 (br d)	71.32
3-OCH_3_	3.84 (s)	55.5
7′-OCH_3_	3.83 (s)	55.2
1′′	4.94 (br s)	101.2
2′′	3.58 (m)	73.4
3′′	3.47 (m)	76.6
4′′	3.58 (m)	69.9
5′′	3.75 (m)	76.2
6′′	3.89 (br d, 5.0)	61.1
	3.75 (m)	

*δ* in ppm and *J* in Hz.

**Table 2:** Effect of Cp extracts on gastric secretion, acidity, and gastric lesion index in pylorus-ligated Shay rats (mean ± SEM).

Treatment	Dose (mg/kg, i.g.)	Volume of gastric content (mL)	Titratable acidity (mEq/L)	Ulcer index
Control (distilled water)	—	10.50 ± 0.18	174.44 ± 4.44	2.83 ± 0.30
Ranitidine	50	3.75 ± 0.21^^*∗∗∗*^^	56.66 ± 3.10^*∗∗∗*^	0.50 ± 0.22^*∗∗∗*^
Cp leaves	200	8.33 ± 0.35^*∗∗∗*^	101.66 ± 5.07^*∗∗∗*^	1.16 ± 0.30^*∗∗*^
Cp leaves	400	5.36 ± 0.22^*∗∗∗*^	97.77 ± 2.93^*∗∗∗*^	0.83 ± 0.30^*∗∗∗*^
Cp fruits	200	10.33 ± 0.24	145.55 ± 4.52^*∗∗∗*^	1.83 ± 0.30^*∗*^
Cp fruits	400	9.78 ± 0.31	144.66 ± 7.19^*∗∗*^	1.16 ± 0.30^*∗∗*^
Cp flowers	200	9.15 ± 0.29^*∗∗*^	156.66 ± 3.10^*∗*^	2.00 ± 0.25
Cp flowers	400	7.46 ± 0.21^*∗∗∗*^	132.22 ± 3.29^*∗∗∗*^	1.30 ± 0.33^*∗∗*^

Six rats were used in each group. ^*∗*^*p* < 0.05, ^*∗∗*^*p* < 0.01, and ^*∗∗∗*^*p* < 0.001 versus control group.

**Table 3:** Effect of Cp extracts on gastric lesions induced by different ulcerating agents (mean ± SEM).

Treatment	Dose (mg/kg, i.g.)	80% EtOH	0.2 M NaOH	25% NaCl
Control (distilled water)	1 ml	7.66 ± 0.21	7.16 ± 0.30	6.50 ± 0.22
Ranitidine	50	1.00 ± 0.25^*∗∗∗*^	0.83 ± 0.30^*∗∗∗*^	0.83 ± 0.30^*∗∗∗*^
Cp leaves	200	2.83 ± 0.30^*∗∗∗*^	2.66 ± 0.33^*∗∗∗*^	2.83 ± 0.30^*∗∗∗*^
Cp leaves	400	2.16 ± 0.30^*∗∗∗*^	1.83 ± 0.47^*∗∗∗*^	1.83 ± 0.30^*∗∗∗*^
Cp fruits	200	6.83 ± 0.09^*∗*^	6.83 ± 0.30	6.33 ± 0.33
Cp fruits	400	6.00 ± 0.30^*∗∗*^	6.33 ± 0.33	6.00 ± 0.36
Cp flowers	200	4.66 ± 0.33^*∗∗∗*^	5.00 ± 0.36^*∗∗∗*^	4.16 ± 0.30^*∗∗∗*^
Cp flowers	400	4.00 ± 0.36^^*∗∗∗*^^	4.33 ± 0.33^*∗∗∗*^	3.16 ± 0.30^*∗∗∗*^

Six rats were used in each group. ^*∗*^*p* < 0.05, ^*∗∗*^*p* < 0.01, and ^*∗∗∗*^*p* < 0.001 versus control group.

**Table 4:** Effect of Cp extracts on indomethacin-induced gastric mucosal lesions (mean ± SEM).

Treatment	Dose (mg/kg, i.g.)	Ulcer index
Control (indomethacin)	30	40.00 ± 2.55
Ranitidine	50	18.50 ± 1.76^*∗∗∗*^
Cp leaves	200	31.83 ± 1.70^*∗*^
Cp leaves	400	21.50 ± 1.02^*∗∗∗*^
Cp fruits	200	37.50 ± 2.20
Cp fruits	400	34.66 ± 1.42
Cp flowers	200	36.83 ± 1.47
Cp flowers	400	31.83 ± 1.24^*∗*^

Six rats were used in each group. ^*∗*^*p* < 0.05, ^*∗∗*^*p* < 0.01, and ^*∗∗∗*^*p* < 0.001 versus indomethacin group.

**Table 5:** Effect of Cp extracts on hypothermic restraint stress-induced intraluminal bleeding and gastric lesions in rats (mean ± SEM).

Treatment	Dose (mg/kg, i.g.)	Intraluminal bleeding score	Gastric lesion ulcer index
Control (distilled water)	—	3.83 ± 0.30	30.83 ± 0.98
Ranitidine	50	0.66 ± 0.21^*∗∗∗*^	9.00 ± 0.73^*∗∗∗*^
Cp leaves	200	2.83 ± 0.30^*∗*^	21.83 ± 1.53^*∗∗∗*^
Cp leaves	400	2.00 ± 0.25^*∗∗∗*^	14.50 ± 0.76^*∗∗∗*^
Cp fruits	200	4.00 ± 0.25	28.50 ± 0.61
Cp fruits	400	3.50 ± 0.22	25.66 ± 1.17^*∗*^
Cp flowers	200	2.66 ± 0.33^*∗*^	24.66 ± 0.42^*∗∗∗*^
Cp flowers	400	2.33 ± 0.21^*∗∗*^	20.50 ± 0.42^^*∗∗∗*^^

Six rats were used in each group. ^*∗*^*p* < 0.05, ^*∗∗*^*p* < 0.01, and ^*∗∗∗*^*p* < 0.001 versus control group.

**Table 6:** *In vitro* cytotoxic activity (IC_50_ values^*∗*^) of Cp extracts and compounds on four human cancer cell lines.

Sample conc. (*µ*g/mL)	MCF-7	HCT-116	HepG-2	A-549
Ethanol leaf extract	98.20 ± 8.32^a^	30.50 ± 1.44^a^	27.40 ± 1.65	41.20 ± 7.04^a^
Ethanol flower extract	68.20 ± 8.54^a^	28.60 ± 1.06^a^	24.50 ± 1.68	207.30 ± 8.50^a^
Ethanol fruit extract	49.70 ± 4.10^a^	55.60 ± 2.61^a^	46.80 ± 3.70^a^	56.90 ± 2.21^a^
**1**	51.40 ± 4.19^a^	58.60 ± 2.46^a^	45.60 ± 3.79^a^	53.0 ± 3.74^a^
**2**	12.60 ± 2.0^a^	6.80 ± 0.46^c^	4.99 ± 0.60^c^	6.03 ± 0.52
**5**	45.70 ± 4.61^a^	11.60 ± 1.30^b^	7.19 ± 0.58^a^	26.60 ± 2.37^a^
**10**	29.20 ± 0.92^a^	21.50 ± 4.85^b^	10.40 ± 0.98^a^	6.50 ± 0.38
**11**	18.0 ± 3.40^a^	6.16 ± 0.99	3.49 ± 0.21	14.30 ± 0.63^a^
Vinblastine sulfate (positive control)	1.65 ± 0.17	5.39 ± 0.43	3.48 ± 0.22	6.05 ± 0.67

^a^ *p* < 0.001, ^b^*p* < 0.01, and ^c^*p* < 0.05 compared to reference drug. ^*∗*^IC_50_: concentration of extract required to reduce cell survival by 50%.

## Additional Points

*Supplementary Data.*  ^1^H NMR, ^13^C NMR, 2D NMR, and ESI-MS data of compound** 1** are available as Supplementary Material, available online at https://doi.org/10.1155/2017/8086791.
