# Supplementary material for: Evaluation of Antiulcer and Cytotoxic Potential of the Leaf, Flower, and Fruit Extracts of Calotropis procera and Isolation of a New Lignan Glycoside
Source: Evid Based Complement Alternat Med. 2017 Aug 30;2017:8086791. doi: 10.1155/2017/8086791 (PMC5603131; doi:10.1155/2017/8086791)
Supplement: Supplementary file 1 — Fig. 1: 13C NMR spectrum of compound 1. Fig. 2: Expanded 13C NMR spectrum of compound 1. Fig. 3: DEPT 135 NMR spectrum of compound 1. Fig. 4: Expanded DEPT 135 NMR spectrum of compound 1. Fig. 5: DEPT 90 NMR spectrum of compound 1. Fig. 6: 1H NMR spectrum of compound 1. Fig. 7: Expanded 1H NMR spectrum of compound 1. Fig. 8: HMBC spectrum of compound 1. Fig. 9: HSQC spectrum of compound 1. Fig. 10: ESI-MS (+ve) spectrum of compound 1. [file 8086791.f1.docx]

Supporting Information

Evaluation of antiulcer and cytotoxic potential of the leaf, flower and fruit extracts of *Calotropis procera* and isolation of a new lignan glycoside

# **Areej Mohammad Al-Taweel,^1^ Shagufta Perveen,^1^*** Ghada Ahmed Fawzy,^1,2^ Attiq Ur Rehman,^3^ Afsar Khan,^3^**** Rashad Mehmood,^4^ Laila Mohamed Fadda^5^**

*^1^Department of Pharmacognosy, College of Pharmacy, King Saud University, Riyadh, P.O. Box 2457, Riyadh 11451, Saudi Arabia*

# *^2^Department of Pharmacognosy, Faculty of Pharmacy, Cairo University, Cairo 11562, Egypt*

# *^3^Department of Chemistry, COMSATS Institute of Information Technology, Abbottabad-22060, Pakistan*

### *^4^Department of Chemistry, University of Education, Vehari Campus, Vehari-61100, Pakistan*

*^5^Department of Pharmacology, College of Pharmacy, King Saud University, Riyadh, P.O. Box 2457, Riyadh 11451, Saudi Arabia*

_________________________

Correspondence should be addressed to Afsar Khan; afsarhej@gmail.com and Shagufta Perveen; [shagufta792000@yahoo.com](mailto:shagufta792000@yahoo.com)

TABLE OF CONTENTS

Fig. 1: ^13^C NMR spectrum of compound 1.

Fig. 2: Expanded ^13^C NMR spectrum of compound 1.

Fig. 3: DEPT 135 NMR spectrum of compound 1.

Fig. 4: Expanded DEPT 135 NMR spectrum of compound 1.

Fig. 5: DEPT 90 NMR spectrum of compound 1.

Fig. 6: ^1^H NMR spectrum of compound 1.

Fig. 7: Expanded ^1^H NMR spectrum of compound 1.

Fig. 8: HMBC spectrum of compound 1.

Fig. 9: HSQC spectrum of compound 1.

Fig. 10: ESI-MS (+ve) spectrum of compound 1.


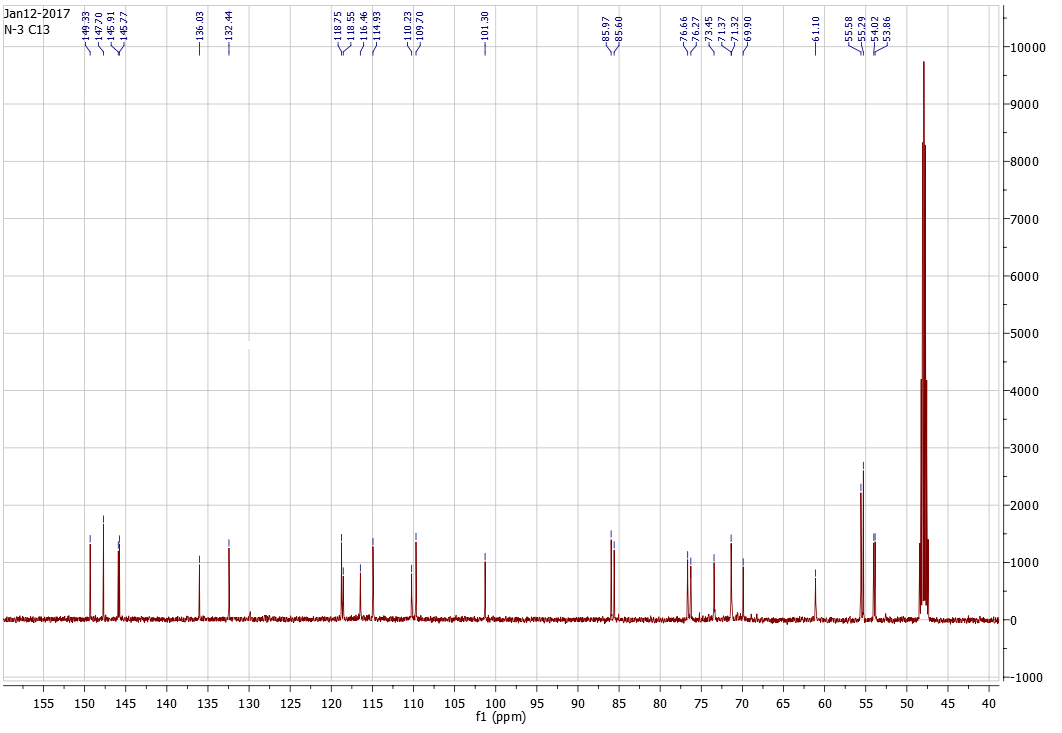


Fig. 1: ^13^C NMR spectrum of compound 1


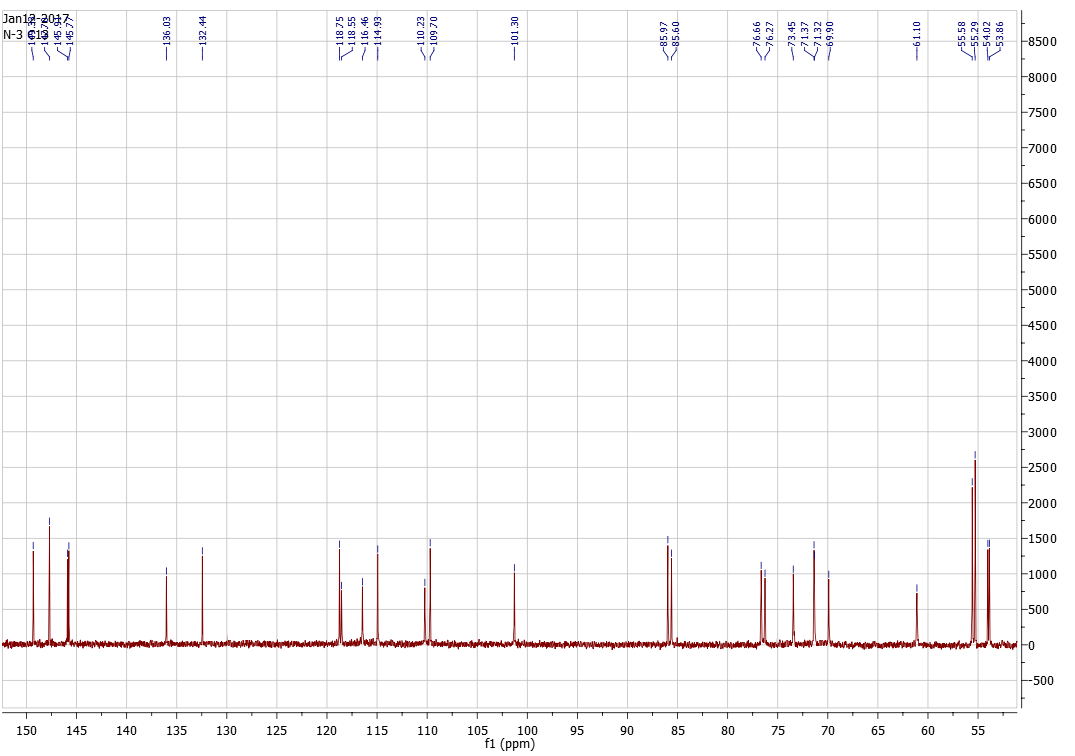


Fig. 2: Expanded ^13^C NMR spectrum of compound 1


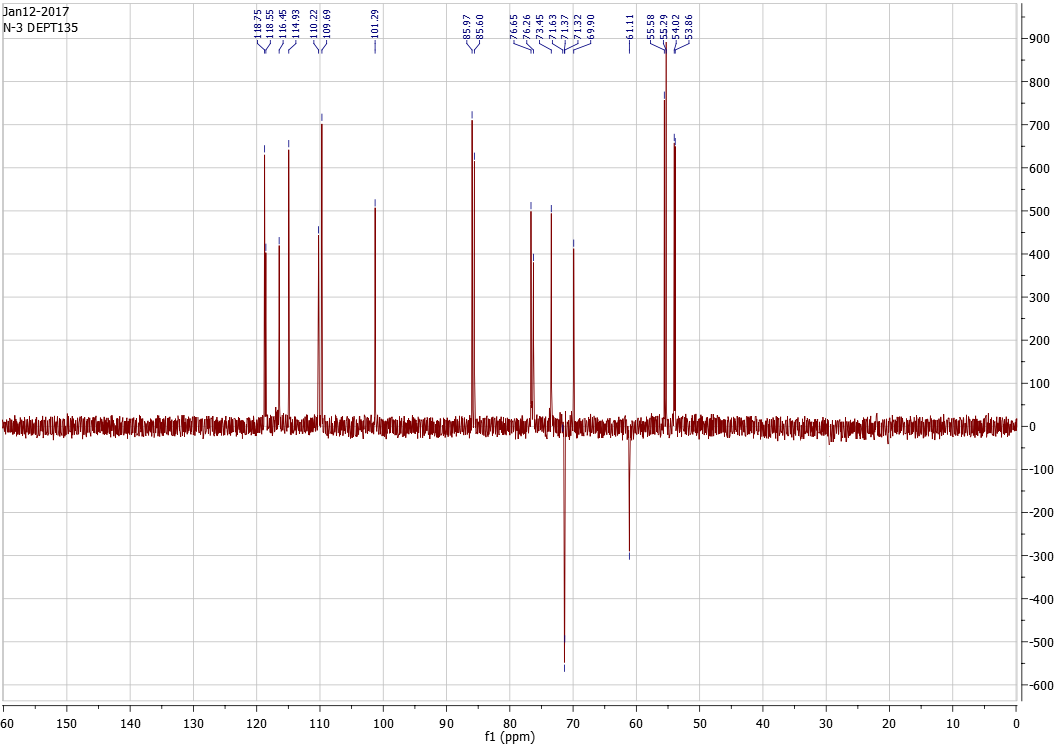


Fig. 3: DEPT 135 NMR spectrum of compound 1


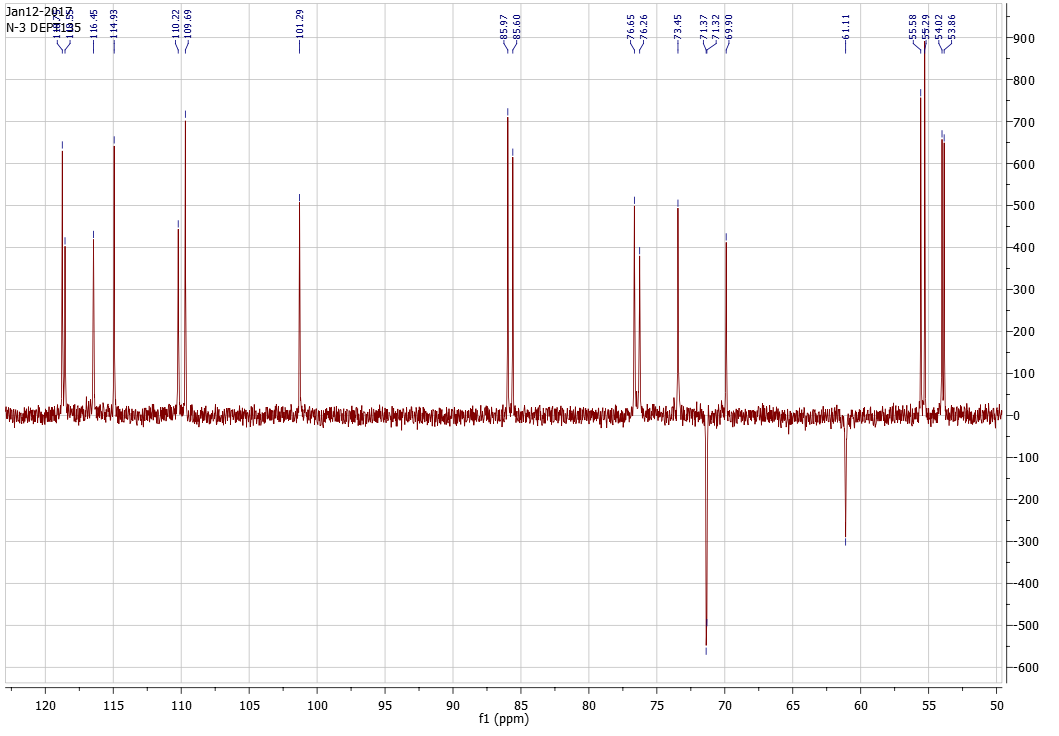


Fig. 4: Expanded DEPT 135 NMR spectrum of compound 1


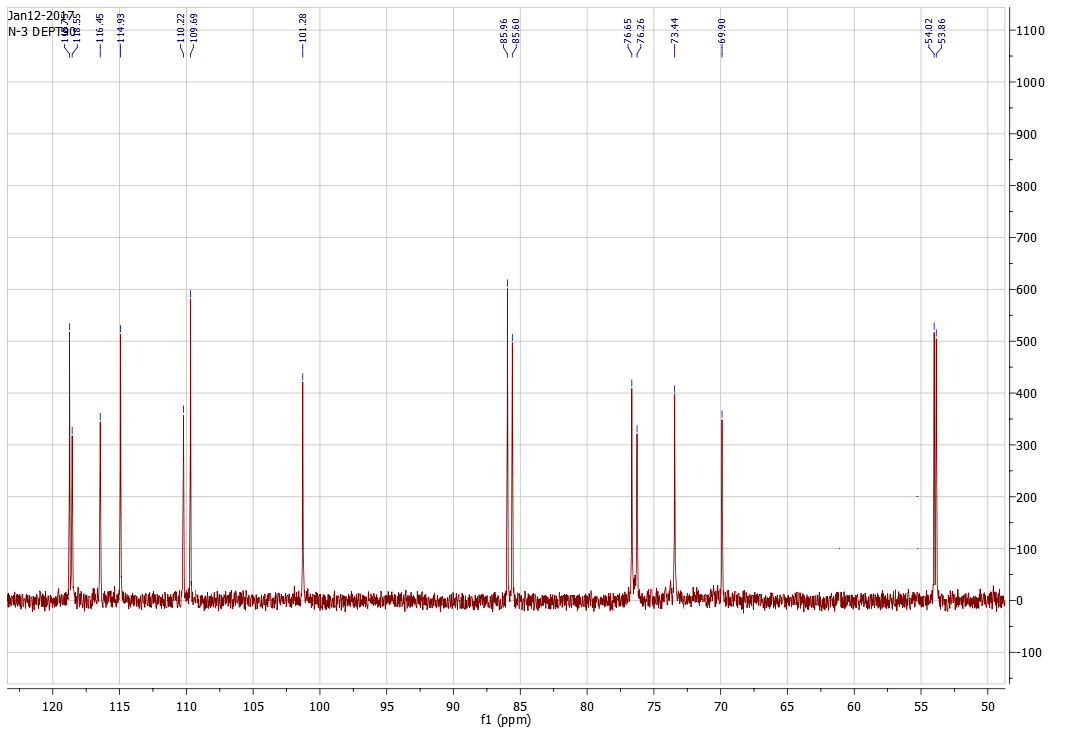


Fig. 5: DEPT 90 NMR spectrum of compound 1


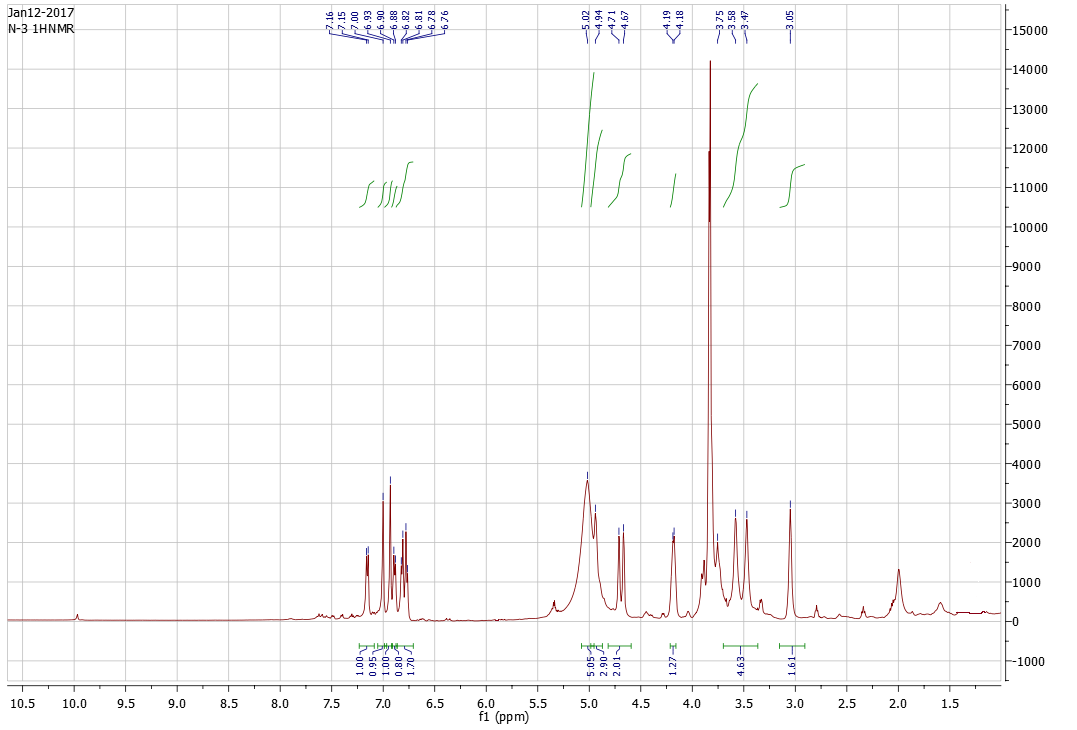


Fig. 6: ^1^H NMR spectrum of compound 1


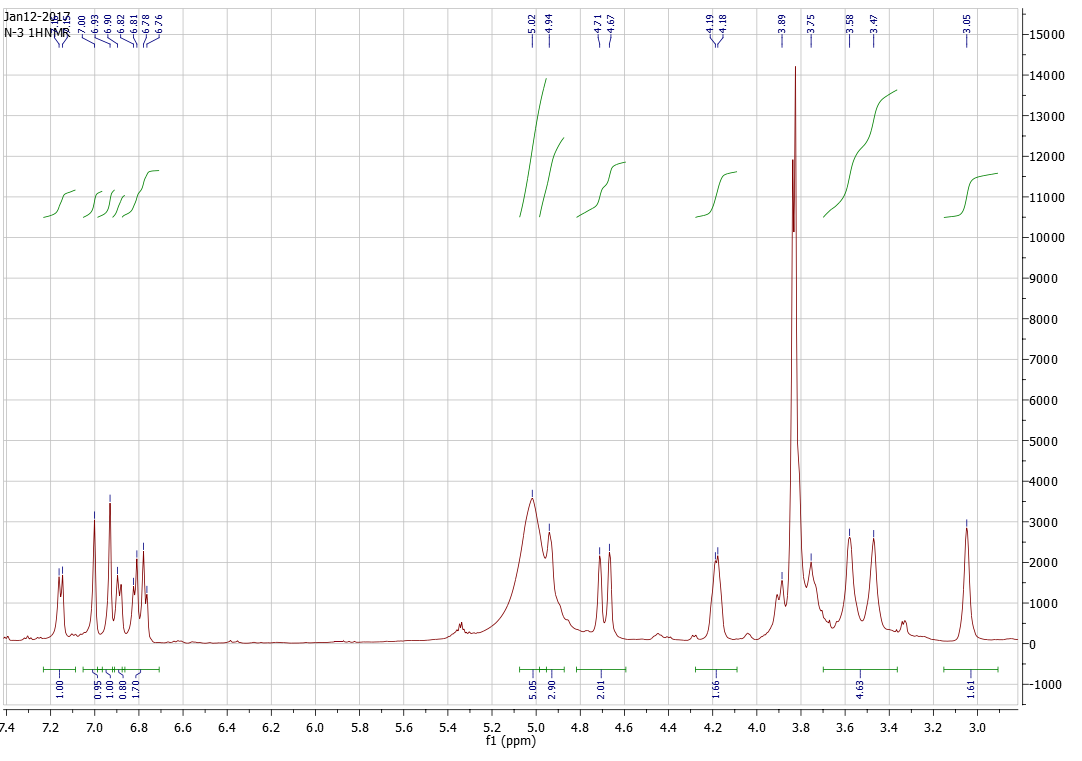


Fig. 7: Expanded ^1^H NMR spectrum of compound 1


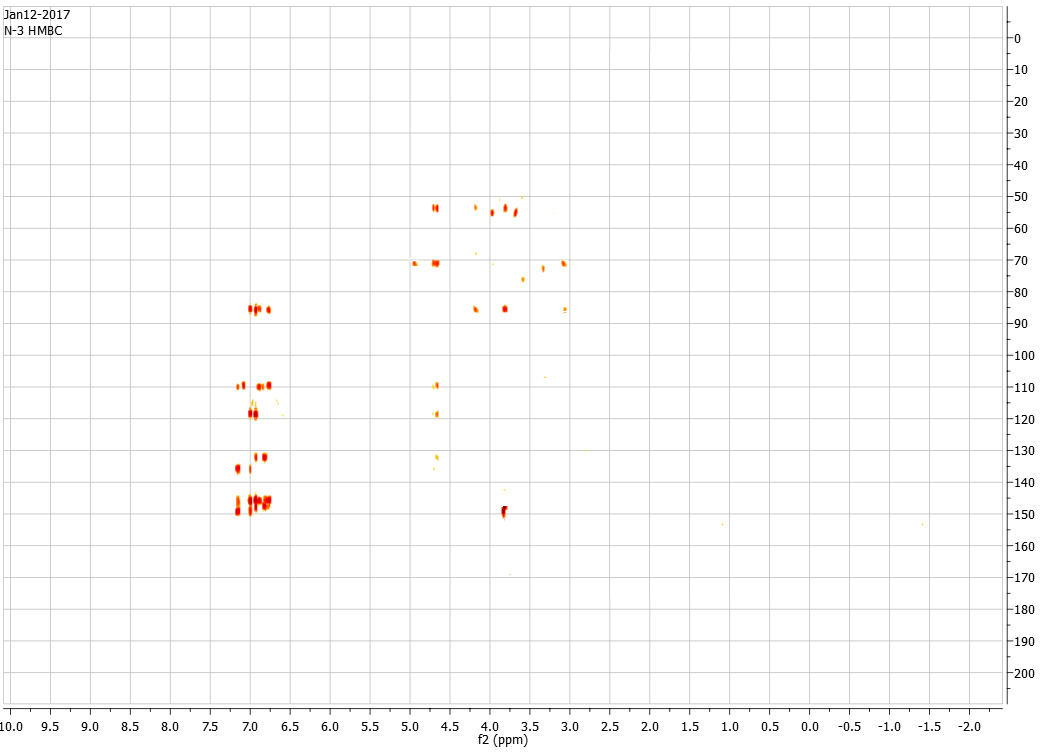


Fig. 8: HMBC spectrum of compound 1


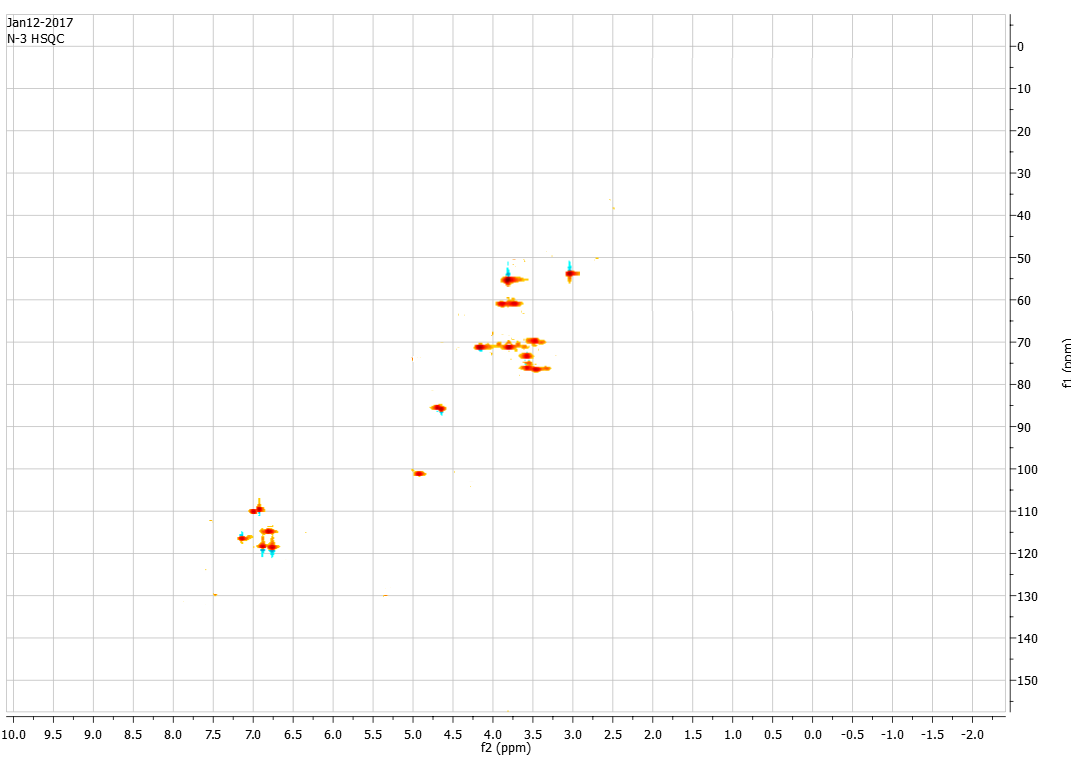


Fig. 9: HSQC spectrum of compound 1


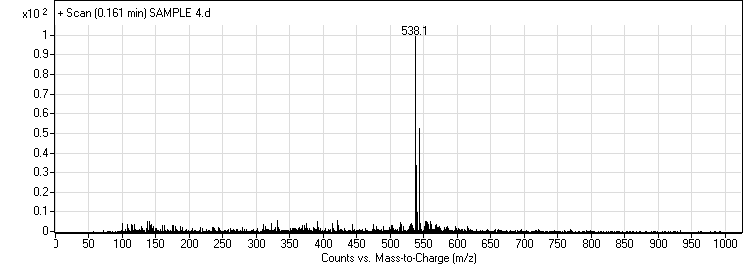


Fig. 10: ESI-MS (+ve) spectrum of compound 1
